# A pilot study of cognitive behavioural therapy and relaxation for migraine headache: a randomised controlled trial

**DOI:** 10.1007/s00415-015-7916-z

**Published:** 2015-10-17

**Authors:** S. Cousins, L. Ridsdale, L. H. Goldstein, A. J. Noble, S. Moorey, P. Seed

**Affiliations:** Kings College London, Denmark Hill Campus, PO57, SE5 8AF London, UK; University of Liverpool, Merseyside, UK

**Keywords:** Migraine, Cognitive behavioural therapy, Relaxation, Headache

## Abstract

Headache is being viewed more commonly in a biopsychosocial framework, which introduces the possible utilisation of psychological treatment options, such as cognitive behavioural therapy and relaxation. No such treatments have been trialled in the UK. We conducted a randomised controlled pilot trial, comparing a brief guided self-help CBT and relaxation treatment with standard medical care (SMC), in a UK NHS setting. Participants were recruited from specialist headache clinics across London. Participants were randomised to receive either treatment or standard medical care. Our objective was to provide design information necessary for a future definitive trial of the SHE treatment, including, recruitment/retention rates, acceptability of randomisation, treatment fidelity and estimations of mean and variances of outcome measures. From the initial 275 patients identified, 73 were randomised. There was no difference in drop-out rates between SMC and treatment groups. Of the 36 participants randomised to receive treatment, 72 % attended all sessions. Findings show that a future definitive trial of the SHE treatment is feasible, with small modifications of protocol, within a UK NHS context.

## Introduction

Migraine headache is the third most prevalent condition [1] and seventh highest among specific causes of disability, globally [2, 3]. In the UK, 18 % of women and 8 % of men suffer from migraine [4]. Personal, social and work life can be severely affected [5], with quality of life, both during and between attacks, substantially reduced [6]. Service and social costs are also high, and increase with symptom severity [7].

Current standard treatment for migraine headache is medication [8]. However, high levels of psychological comorbidity [9] has led to migraine becoming more commonly viewed as a biopsychosocial condition [10], influenced by cognitive, emotional and environmental factors, as well as biological. Of the 4 % of people who consult their family doctor for headache, 28 % have clinically significant levels of anxiety or depression [11]. Headache is the most common reason for referral to neurology [12], with those referred showing higher levels of anxiety about their headache symptoms and consulting more frequently than patients managed in primary care [11]. Lifestyle factors, such as stress and sleep quality, can contribute significantly to onset and course of headaches, and management of these factors, including identification of environmental triggers, is increasingly important in the treatment of migraine. Viewing headache in a biopsychosocial framework introduces the possible utilisation of psychological treatment options, such as cognitive behavioural therapy (CBT), relaxation and biofeedback. These treatments allow patients to develop preventative and acute management strategies, such as trigger identification, modification of maladaptive inter-related thoughts, feelings and behaviours surrounding headache, as well as physiological auto-regulation strategies. Reviews of randomised controlled trials assessing the effectiveness of behavioural treatments found a 32 % reduction in headache frequency compared to 5 % in controls [13]. Furthermore, Holroyd et al. [14] found that combining a behavioural treatment programme with prophylactic drug treatment was more effective in reducing migraine frequency than either of the two treatments presented on their own. Relaxation and CBT treatments when delivered separately have been found to show similar effectiveness in treating headache [15], and minimal contact [3, 4] sessions, combined with written material and audiotapes, can achieve outcomes as good as more intensive and expensive treatments [16, 17].

The effectiveness of psychological treatments has been shown primarily by studies carried out in the United States and in some European countries (US) [15, 16]. In the US health systems, heavy reliance on private insurance funding and likely cost barriers to recruitment to this treatment limits the generalizability of these studies. Even in countries where universal care has been provided for many years, the population profile and systems of delivery are different, making it difficult to generalise findings from complex interventions. The United Kingdom’s (UK) National Health Service (NHS), a publically funded health system, provides universal access in principle to all socio-economic groups [18]. No such treatments have been developed and tested in the UK, tailored to the NHS context. In the context of this research gap, the UK’s National Institute for Health and Care Excellence (NICE) called for studies investigating the effectiveness of psychological treatments for headache [8].

Currently, it is unknown whether a full RCT assessing a non-drug psychological treatment for headache would succeed in terms of recruitment and retention rates in the UK. MRC guidelines for developing and evaluating complex interventions highlight the importance of the piloting of complex interventions and warn against a focus solely on evaluation, to the detriment of adequate development and pilot work [19]. Indeed, only 1 in 3 MRC and NIHR trials have been found to recruit their target sample in time [20], underlining the need for pilot trials that provide insight into the ability to recruit and retain participants, as well as increasing the likelihood of a high-quality definitive RCT.

In line with MRC guidelines, we conducted a pilot trial to provide design information necessary for a future definitive RCT, which could assess the effectiveness of the SHE treatment. We were informed by Lancaster et al. [21] in defining our objectives. As recommended, the significance of treatment effect was not assessed in this pilot study. Our pilot trial objectives were i) to calculate recruitment, consent and follow-up rates, ii) to test acceptability of randomisation to participants, iii) to assess treatment fidelity of SHE, and iv) to provide estimates of the mean and standard deviation of the outcomes measures to inform future sample size calculation.

## Method

Participants were recruited from specialist headache clinics (neurologists and family doctors with a special interest in headache) across London. Participants who expressed an interest in the study were invited to undergo a screening assessment. Inclusion criteria were adults (men and women) aged 18–75 years; diagnosis of migraine headache; onset >6 months previously; and >3 headache days per month (assessed by headache diary; including both episodic and chronic migraine). Exclusion criteria were physical conditions likely to cause headache (secondary headache); pregnancy; current psychotic illness; substance dependency (not including headache rescue medication); currently undergoing psychological therapy; and inability to complete self-report measures. Patients were recruited according to the criteria available at the time which was the HIS classification guidelines (2nd Edition). Patients with migraine without and with auras were included. Additional diagnosis of medication overuse headache was not an exclusion criterion. Suitable participants completed outcome measures at a baseline (face-to-face) assessment, carried out either at the participants’ home or the research site, and were then randomised to SMC or treatment (plus SMC) groups. Trialists waited until any changes to prophylactic medication had been implemented and stabilised before recruitment. Participants were followed up at 2 and 4 months and were given a £20 shopping voucher on completion of the final follow-up assessment. Ethical approval was obtained from South East London regional ethics committee (10/H0805/79) and informed consent was obtained from all participants. The trial is ISRCTN registered (ISRCTN53460881) and is on the UK Clinical Research Network Study Portfolio (UKCRN ID 11265).

### Treatment

The treatment combines CBT and relaxation (deep breathing and progressive muscle relaxation) training in a minimal contact manualised intervention, carried out over 5 weeks in three face-to-face sessions, alternating with two telephone calls. The treatment was delivered by one trained [Improving Access to Psychological Therapies (IAPT)] CBT therapist. The therapist received a therapist’s manual (available from LR), containing details regarding delivery and content of the treatment, and underwent bi-weekly supervision sessions with a senior CBT therapist SM. The therapists’ manual was previously developed by LR, and feedback from users incorporated. Participants were given a copy of a patient manual, developed and structured to be used alongside and add to the therapy sessions. Session 1 (week 1) introduced the concept of links between thoughts, feelings, symptoms and behaviours, thought monitoring and relaxation techniques. Participants were given headache and thought diaries to complete at home and were asked to practice relaxation techniques (with an accompanying CD) for approximately 15 min each day (recording levels of tension before and after). Session 2 (week 3) introduced problem solving and cognitive restructuring techniques, including alternative thinking. Session 3 (week 5) built on alternative thinking techniques and covered relapse prevention. Thought and headache diaries were reviewed in sessions 2 and 3, and phone calls (weeks 2 and 4) were used to review practice and identify any difficulties. The patient manual includes an educational component where patients are given information about what migraine is, medication, as well as informing them about identifying and managing triggers, including stress. The average duration of face-to-face sessions was 63 min (range 34–96). Participants randomised to the SMC continued treatment as usual with no restrictions, and received the patient handbook in the post after the final follow-up assessment.

### Fidelity measure

On the basis of the therapist and patient manuals, a checklist of treatment components was devised. Eight rating scales (1 = very poor to 7 = excellent) assessing therapeutic skill (e.g. supportive encouragement, communication style, overall therapeutic alliance) were also included. Two CBT therapists unconnected with the trial completed the fidelity measure. They were given audio-recordings of 30 randomly selected face-to-face treatment sessions (*n* = 10 each of sessions 1, 2 and 3), and asked to identify whether each treatment component was present in the session and rate the level of therapeutic skill on each of the 8 rating scales. The fidelity measure was piloted on two randomly selected sessions to clarify the meaning of individual items and for raters to improve the clarity of coding instructions.

### Outcome measures

All outcome measures (self-report questionnaires) were completed at baseline and four months (primary end-point). In order to enhance retention, participants also carried out a shortened assessment at 2 months, by post. Primary outcome measure was number of headache days, assessed by headache diary completed during the months (28 days) immediately preceding baseline and four-month follow-ups. Participants were asked to record in the diary the date, duration and pain intensity (0 = no pain, to 10 = worst possible pain) of each headache experienced, as well as any rescue medications taken at the time of the headache. A headache day was defined as a day containing 2 or more hours of headache. Secondary outcome measures included Migraine Disability Assessment (MIDAS) [22], assessing migraine related disability, a higher score reflecting more severe disability (>21 = severe disability); Headache Impact Test [23] (HIT-6), assessing the impact of headache, score range is 36–78, with a higher score reflecting higher impact; Hospital Anxiety and Depression Scale (HADS) [24], a state-based measure of anxiety and depression, where for each subscale a score of ≥11 shows probable presence of a mood disorder [25]; Brief Illness Perceptions Questionnaire (B-IPQ) [26], assessing the degree to which headaches are perceived as threatening or benign, a higher score reflects a more threatening view of headache; and EuroQol (EQ-5D) [27], assessing health-related quality of life across five dimensions (mobility, self-care, usual activities, pain/discomfort, anxiety/depression).

### Randomisation and blinding

Randomisation was carried out using a web-based Independent Randomisation Service provided by King’s College London, Clinical Trials Unit (KCL, CTU). The researcher requested randomisation by logging in with a unique username and password, and entering coded participant details. Participants were allocated to either SMC or treatment group using this system. Emails were automatically generated and sent to the researcher (blinded) confirming randomisation and to the therapist (unblinded) giving randomisation details. The randomisation method used was minimisation, with minimisation factor—frequency of headache days (1. ≤14 headache days per month 2. ≥15 headache days per month).

Participant assessments and data entry were carried out by a researcher (SC) blinded to treatment allocation. At assessments and during treatment sessions (if in treatment group) participants were actively reminded that the researcher should not be made aware of treatment allocation. Outcome measure data were entered by the researcher into the Online Data Capture and Management Service, MACRO database (developed by Infermed, provided by KCL CTU), which is able to securely capture data online, via bespoke electronic case report forms. For the purposes of analysis, the researcher was unblinded once the data for all follow-ups had been securely entered to the MACRO system.

### Sample size calculation

We considered that 30 patients in each arm of the study would provide estimates of the properties of the main outcome measure with adequate precision to be able to calculate the sample size needed for a definitive trial. Sixty participants would allow estimation of the standard deviation (SD) to within 20 % of the true value (based on the Chi-square distribution). The use of the upper one-sided 80 % interval guarded against the study failing due to inadequate power at a cost of only a 16 % greater sample size. The correlation between repeated measures can likewise be estimated to within under 0.1 units of the true value (upper one-sided 80 % interval). Estimating a 15–25 % drop-out rate at 6 months, we therefore planned to recruit 40 patients in each arm to allow for possible discontinuation, making 80 patients in total.

### Statistical analysis

We calculated key information relating to our objectives, including, uptake, eligibility, recruitment and retention rates, and treatment attendance. Baseline demographics were compared between completer and non-completer groups using independent samples t-tests and Chi-square tests for categorical variables. All analyses were based on the intention to treat principle.

## Results

### Participant recruitment and retention

**Fig. 1:**
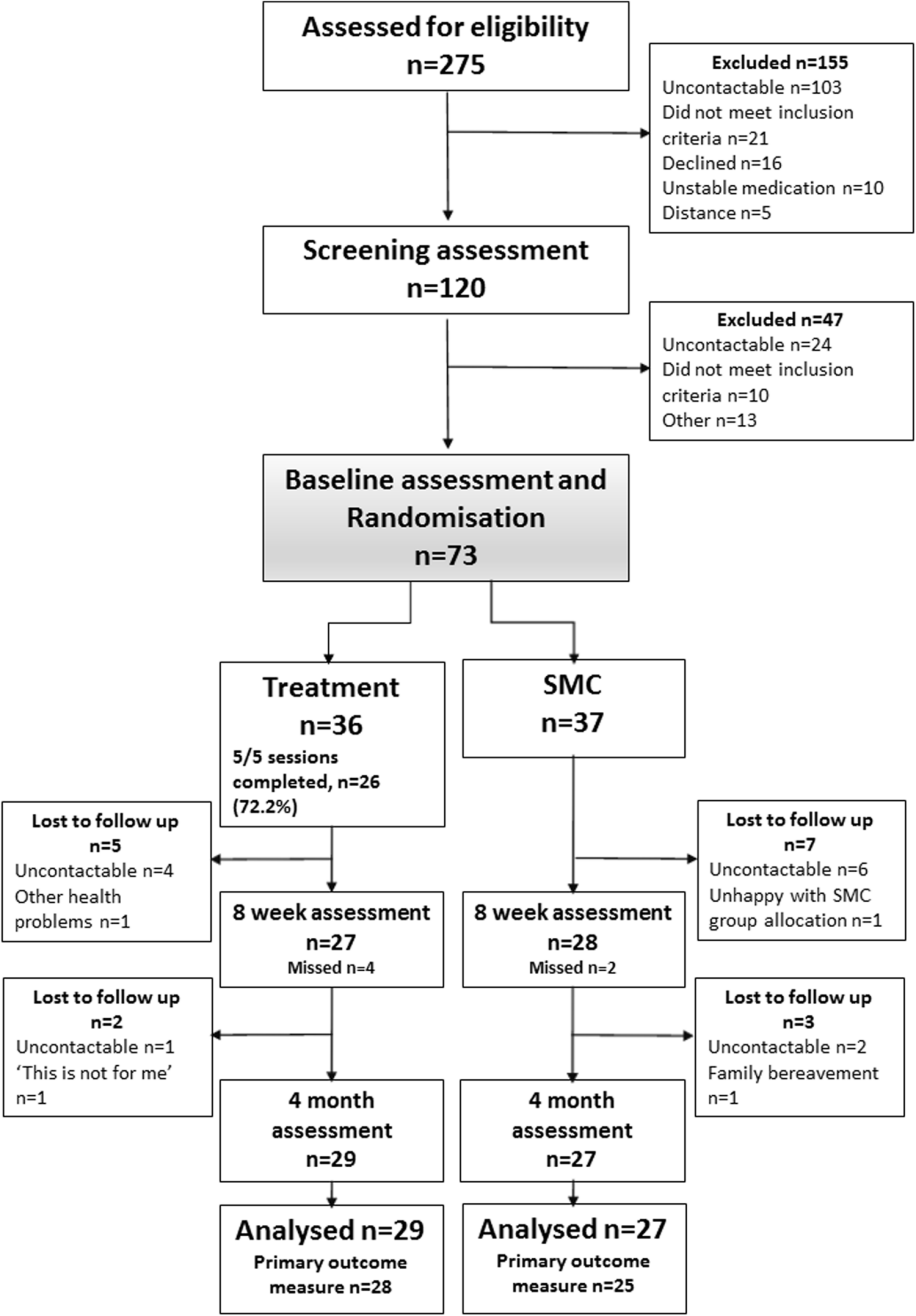
Diagram showing participant flow through the study. Excluded after screening assessment—‘other’ reasons include personal reasons (*n* = 4); pursuing other treatment (*n* = 3); relocation (*n* = 1); health reasons (*n* = 2); unstable medication (*n* = 2); no reason given (*n* = 1). ‘Did not meet inclusion criteria’ reasons include insufficient headache days (*n* = 7), abnormal MRI scan (*n* = 2), in psychological therapy (*n* = 1)

Figure 1 illustrates participant flow through the study. From the initial 275 patients identified, between August 2012 and March 2014, 120 consented to a screening assessment and 73 participants were then fully recruited into the trial. These participants completed a baseline assessment and were randomised to receive either SMC (*n* = 37) or the treatment (*n* = 36). At baseline assessment, the two groups (SMC and treatment) were similar (Table 1).

**Table 1:** Baseline measures for SMC and Treatment groups

Baseline measures	ALL	SMC (*n* = 37)	Treatment (*n* = 36)	Difference (SMC vs intervention)	95 % confidence interval	
					Lower	Upper
Gender						
Female (%)	60 (82.2)	32 (86.5)	28 (77.8)			
Male (%)	13 (17.8)	5 (13.5)	8 (22.2)			
Age (range, years), sd	39 (19–71)	37.97 (19–71), 12.04	40.67 (19–70), 12.79	2.70	−2.61	3.96
Ethnicity						
Asian (%)	1 (1.4)	0 (0)	1 (2.8)			
Black (%)	12 (16.4)	9 (24.3)	3 (8.3)			
White (%)	53 (72.6)	24 (64.9)	29 (80.6)			
Mixed (%)	5 (6.8)	2 (5.4)	3 (8.3)			
Other (%)	1 (1.4)	1 (2.7)	0 (0)			
Mean diary headache days (sd)	11.780 (7.67)	11.54 (6.64)	12.03 (8.70)	0.49	−0.43	3.67
Mean number of days rescue medication used in diary month (sd)	6.89 (5.55)	7.08 (5.87)^a^	6.69 (5.30)	−0.39	0.43	3.39
MIDAS, mean score (sd)	58.51 (45.57)	65.78 (46.79)	51.023 (43.68)	−14.75	15.08	39.00
HIT-6, mean score (sd)	66.23 (5.16)	65.97 (4.41)	66.5 (5.88)	0.53	−0.49	2.26
HADS-anxiety, mean score (sd)	8.56 (3.84)	9.32 (3.55)	7.78 (4.01)	−1.54	1.57	3.57
HADS-depression, mean score (sd)	5.75 (3.89)	5.68 (3.09)	5.83 (4.61)	0.15	−0.12	1.96
Brief-IPQ, mean score (sd)	52.10 (9.69)	51.41 (9.77)	52.81 (9.69)	1.40	−1.33	3.82

^a^ *n* 1 missing data, *MIDAS* migraine disability assessment scale, *HIT-6* headache impact test, *HADS-A* hospital anxiety and depression scale, Anxiety subscale, *HADS-D* hospital anxiety and depression scale, depression subscale, *Brief-IPQ* Brief Illness Perceptions Questionnaire

**Fig. 2:**
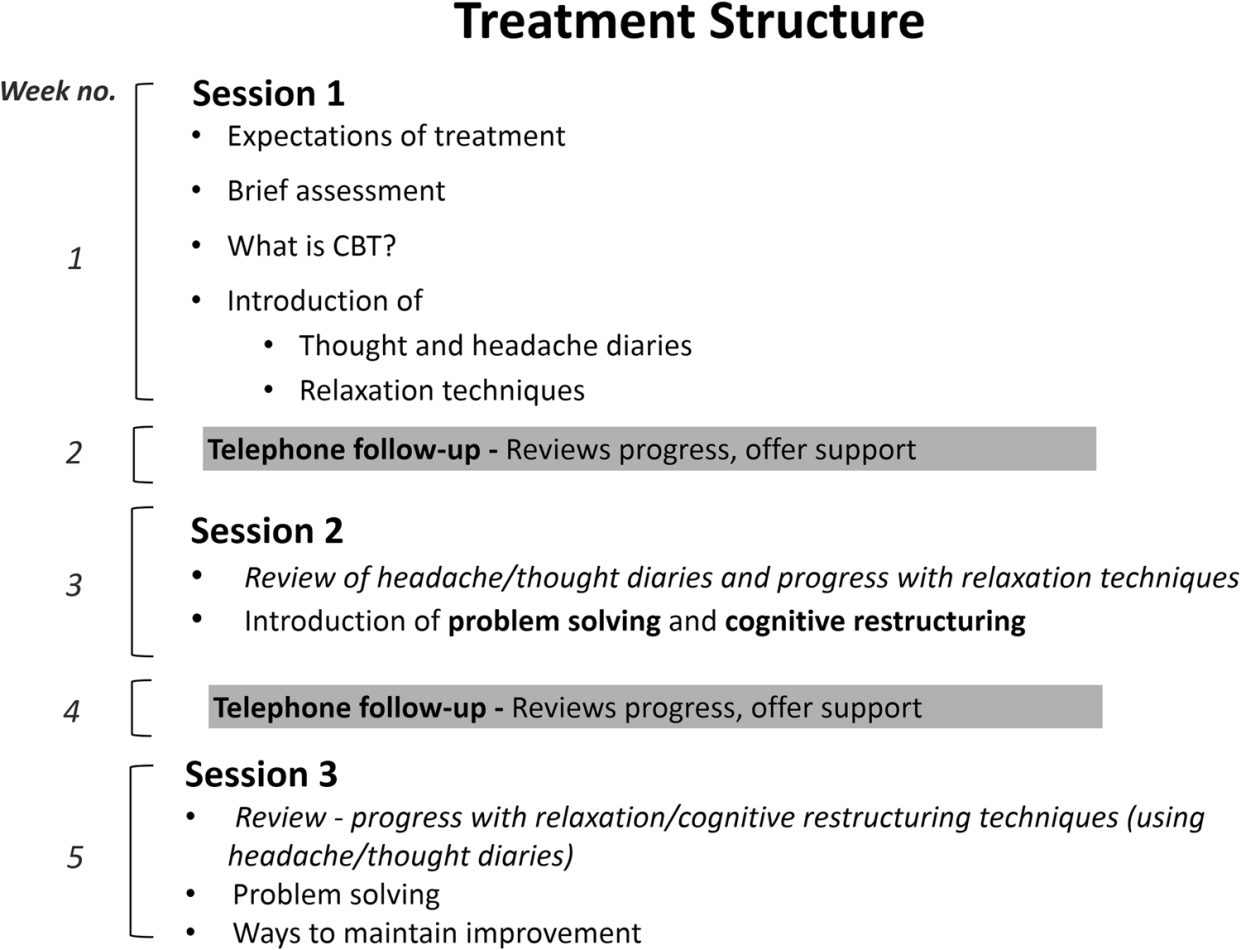
Figure showing timeline and components of the behavioural therapy and relaxation treatment

Fifty-six (76.71 %) participants completed the final four-month follow-up assessment, 27 in the SMC group and 29 participants in the treatment group. Intention to treat analysis was conducted on these participants who had completed the final 4-month follow-up assessment, regardless of receipt of treatment. Primary outcome (Number of headache days) data were obtained for 25 participants in the SMC group and 28 participants in the treatment group and (*n* = 2 and 1, respectively, excluded due to incomplete headache diary). Secondary outcome measures were completed by all participants followed up at the 4-month follow-up (Fig. 2).

The overall loss to final follow-up was 17 (23.29 %), 10 in the SMC group and 7 participants in the treatment group. The loss to follow-up was not significantly different between groups (*X*^2^(1) = 0.59, *p* = 0.44). Table 2 shows no significant difference in the baseline characteristics between completers and non-completers, although there was a trend for non-completers to score higher on the headache-related disability measures at baseline.

**Table 2:** Baseline measures in completer and non-completer groups

Baseline measures	Completers of 4-month assessment (*n* = 56)	Non-completers of 4-month assessment (*n* = 17)	Difference (completers vs non-completers)	95 % confidence interval		*t*	*p*
				Lower	Upper		
Gender							
Female (%)	46 (82.1)	14 (82.4)					0.98
Male (%)	10 (17.9)	3 (17.6)					
Age (range, years), sd	39.96 (19–71), 13.13	37.12 (19–53), 9.64	−2.84	2.93	9.53	0.97	0.33
Ethnicity							
Asian (%)	1 (1.8)	0 (0)					0.11
Black (%)	7 (12.5)	5 (29.4)					
White (%)	42 (75)	11 (64.7)					
Mixed (%)	5 (8.9)	0 (0)					
Other (%)	0 (0)	1 (5.9)					
Mean diary headache days (sd)	11.3 (7.59)	13.35 (7.96)	2.05	−1.98	2.94	−0.94	0.35
Mean number of days rescue medication used in diary month (sd)	6.46 (5.44)	8.38 (5.86)	1.92	−1.87	1.74	−1.20	0.23
MIDAS, mean score (sd)	54.46 (45.64)	71.82 (44.06)	17.37	−16.98	10.81	−1.41	0.16
HIT-6, mean score (sd)	66.20 (5.50)	66.35 (3.98)	0.15	−0.11	2.63	−0.12	0.90
HADS-Anxiety, mean score (sd)	8.29 (3.91)	9.47 (3.54)	1.18	−1.15	1.12	−1.17	0.24
HADS-Depression, mean score (sd)	5.61 (4.07)	6.24 (3.29)	0.63	−0.60	1.58	−0.65	0.52
Brief-IPQ, mean score (sd)	51.70 (9.55)	53.41 (10.33)	1.71	−1.62	4.73	−0.61	0.55

*n* 1 missing data, *MIDAS* migraine disability assessment scale, *HIT-6* headache impact test, *HADS-A* hospital anxiety and depression scale, Anxiety subscale, *HADS-D* hospital anxiety and depression scale, depression subscale, *Brief-IPQ* brief illness perceptions questionnaire

### Unblinding

Unblinding occurred for 22 of the 73 (30.1 %) participants. The majority (*n* = 17) occurred for participants in the treatment group due to, for example, the blinded researcher being contacted about treatment (*n* = 10). Five instances of unblinding occurred in the SMC group with reasons including, participants’ expression of dissatisfaction with treatment group allocation, and communication from a participant regarding receiving the treatment handbook in the post after final follow-up.

### Adherence to treatment protocol

Of the *n* = 36 patients randomly assigned to receive the intervention, 26 (72.2 %) completed all five sessions. Two patients (5.6 %) did not attend any sessions, 3 (8.3 %) completed one session, 1 (2.8 %) completed two sessions, 1 (2.8 %) completed three sessions and 3 (8.3 %) participants completed four sessions.

### Fidelity measure

An average (across raters) of 84 % of components identified in the fidelity checklist were rated as being present by the two raters, in the 30 sessions sampled; session 1—16/17 components present, session 2—9/11 components present, session 3—12/14 components present. The average rating of overall therapeutic reliance across both raters and all sessions was 5 (good).

### Sample size calculation—future definitive trial

Table 3 shows means and variances for all outcome measures, for treatment and SMC groups at baseline and 4-month follow-up. Based on this data, we estimate the effect of the treatment on the main outcome as between −3.2 and +2.4 headache days. We also observed a standard deviation of 6.76, and a correlation of 0.67 between baseline and final outcome. Complete data on 133 patients per group (266 in all) would give 90 % power to detect such a treatment effect. Assuming 76.7 % completion rate (as in the pilot), 347 participants would need to be recruited. The upper 95 % limit for the standard deviation is 8.37. Using this conservative approach about the true SD value, complete data on 203 participants per group (406 in all) would be needed for 90 % power, implying 529 participants to be recruited.

**Table 3:** Baseline and 4-month follow-up assessment outcome measures

	SMC	Treatment	Adj. mean difference	Adj. 95 % CI	
				Lower	Upper
Mean diary headache days (sd)					
Baseline assessment	11.54 (6.64) *n* = 37	12.03 (8.70) *n* = 36			
4-month assessment	9.68 (6.28) *n* = 25	9 (7.27) *n* = 28	−0.45	−3.25	2.40
Mean number of days rescue medication used in diary month (sd)					
Baseline assessment	7.08 (5.87) *n* = 37	6.69 (5.30) *n* = 36			
4-month assessment	6.2 (4.86) *n* = 25	5.86 (5.12) *n* = 28	0.04	−2.38	2.47
MIDAS, mean score (sd)					
Baseline assessment	65.78 (46.79) *n* = 37	51.03 (43.68) *n* = 36			
4-month assessment	53.85 (78.49) *n* = 27	33.86 (34.93) *n* = 29	−15.89	−47.42	15.64
HIT-6, mean score (sd)					
Baseline assessment	65.97 (4.41) *n* = 37	66.5 (5.88) *n* = 36			
4-month assessment	60.85 (8.4) *n* = 27	59.17 (8.19) *n* = 29	−2.00	−6.26	2.25
HADS-Anxiety, mean score (sd)					
Baseline assessment	9.32 (3.55) *n* = 37	7.78 (4.01) *n* = 36			
4-month assessment	7.96 (4.37) *n* = 27	5.76 (4.45) *n* = 29	−1.26	−3.04	0.52
HADS-depression, mean score (sd)					
Baseline assessment	5.68 (3.09) *n* = 37	5.83 (4.61) *n* = 36			
4-month assessment	4.52 (3.51) *n* = 27	4.24 (4.6) *n* = 29	−0.53	−2.40	1.34
Brief-IPQ, mean score (sd)					
Baseline assessment	51.41 (9.77) *n* = 37	52.81 (9.69) n = 36			
4-month assessment	45.26 (10.17) *n* = 27	44.17 (15.89) n = 29	−1.52	−7.88	4.85

*MIDAS* migraine disability assessment scale, *HIT-6* headache impact test, *HADS-A* hospital anxiety and depression scale, Anxiety subscale, *HADS-D* hospital anxiety and depression scale, Depression subscale, *Brief-IPQ* brief illness perceptions questionnaire

## Discussion

This is the first UK-based pilot trial of a minimal contact CBT with relaxation treatment for migraine headache. Findings show adequate recruitment and good treatment adherence. This pilot study did not aim, nor was it powered sufficiently, to comment on the effectiveness of the treatment; however, our results show that a future definitive trial in the UK is feasible.

With regard to patient recruitment, it was originally assumed that 75 % of patients identified would decline participation; our recruitment rates show that of the 275 patients identified, 44 % agreed to undergo a screening assessment and 61 % of those participants screened were consented into the trial. Inability to contact patients after their initial expression of interest was the main reason for patient exclusion before screening, and this may be reduced in a future trial by incorporating the screening assessment into the initial meeting with patients.

Participant adherence to treatment was good. Over 70 % of participants randomised to the treatment group completed all five treatment sessions, with only two participants not attending any sessions.

Participant drop-out rates did not differ between treatment and SMC groups, suggesting that participants accepted randomisation well; however, one participant was unhappy that they had not received treatment and had been allocated to the SMC group, and refused further data collection.

### Treatment fidelity

The fidelity measure and therapeutic alliance scale developed herewith provides a valuable verification of whether treatment is delivered as intended [28], and also enables the evaluation of the role of process factors in predicting outcome. Accurate monitoring of treatment fidelity can increase the internal and external validity of the treatment, allowing more accurate conclusions regarding treatment efficacy in a future definitive trial [29].

Adherence to the treatment protocol may be maximised by ongoing training of those delivering the treatment. Therapists may also be asked to complete treatment component checklists after each session. The application of these fidelity strategies in a future definitive trial, in addition to treatment manuals, can maximise standardisation of treatment delivery [28].

### Limitations and future trial considerations

The current study was not double-blinded; it was not possible to blind the participants or the therapist delivering the treatment to treatment group allocation and participants, once aware of whether they are receiving treatment or not, may value their outcomes differently [30]. This is a common limitation in behavioural intervention RCTs [30] due to the lack of suitable inactive control options. The single-blinding of the researcher collecting outcome data was moderately successful. Accidental unblinding may be reduced in a future trial by—(i) outcome measures being completed through an online or postal submission process, rather than face-to-face, (ii) different researchers undertaking assessments and inputting data, (iii) employment of an unblinded researcher to answer participant and clinician queries, as well as help in the implementation of any nested qualitative studies. These changes would limit the interactions between participants and those researchers assessing outcomes and may reduce accidental unblinding. Participant dissatisfaction with control group allocation may also be reduced in a future trial by patients receiving treatment after final follow-up.

The present study has documented treatment session attendance; however, measuring the degree to which participants perform treatment-related behaviour and cognitive skills in real life settings [31] and amount of practice completed by participants outside of the treatment sessions would be valuable in documenting process measures that may be related to outcome. It is valuable to not only document treatment delivery but also receipt and enactment of treatment by participants [31, 32]. These measures would complement process measures included in the therapeutic alliance scale developed during the current study.

## Conclusion

Previous studies show CBT and relaxation can improve headaches by 49 and 32 %, respectively [33]. Our pilot trial has provided necessary data for sample size calculations for a future trial. It illustrates that a definitive trial of self-guided CBT with relaxation treatment is feasible within the context of the NHS in the UK. Small modifications to the protocol, as described above, will enhance the rigour of a future definitive trial.

## Article highlights

- Studies show minimal contact CBT and relaxation treatments for headache are equally effective.
- This is the first randomised controlled pilot study comparing a combination of a brief guided CBT and relaxation to standard medical care (SMC), in the UK, publicly funded context.
- Findings show adequate recruitment and good treatment adherence.
- A definitive trial of self-guided CBT with relaxation treatment is feasible within the context of the UK NHS.
